# An Unusual Case of Countless Biliary Stones

**DOI:** 10.14309/crj.0000000000000629

**Published:** 2021-07-14

**Authors:** Tarek Nammour, Manar Shmais, Assaad Soweid

**Affiliations:** 1 Division of Gastroenterology, American University of Beirut, Beirut, Lebanon

## CASE REPORT

**Figure 1.**
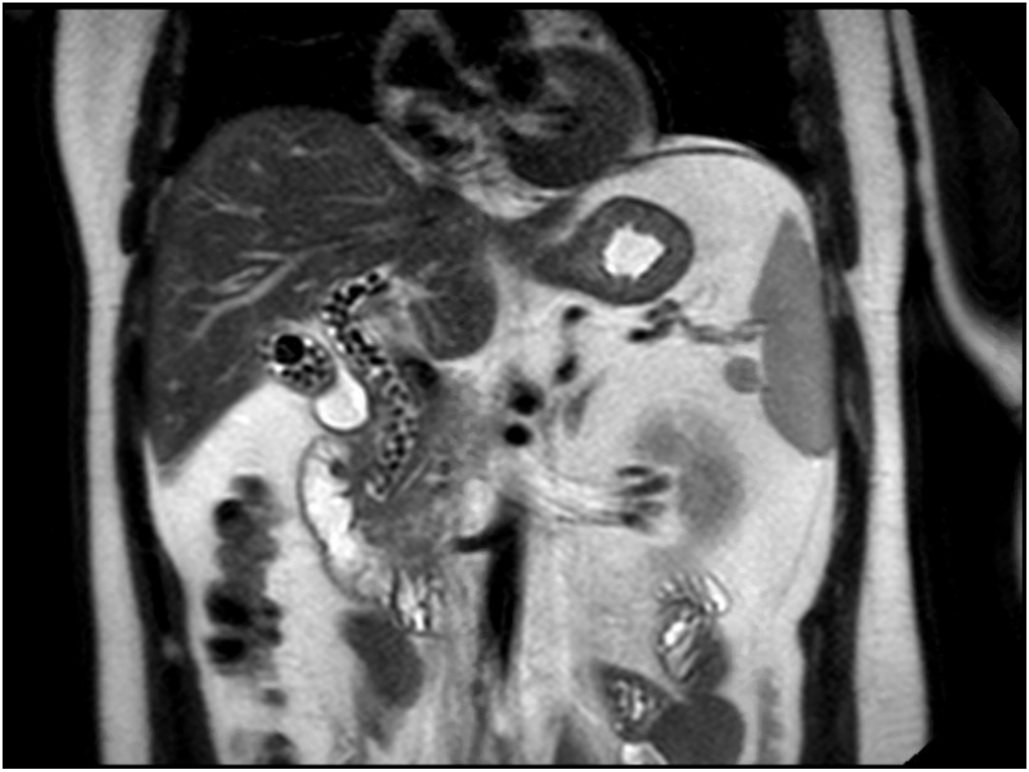
Magnetic resonance cholangiopancreatography showed the gallbladder, dialated common bile duct, common hepatic duct, and proximal right and left hepatic ducts packed with gallstones.

**Figure 2.**
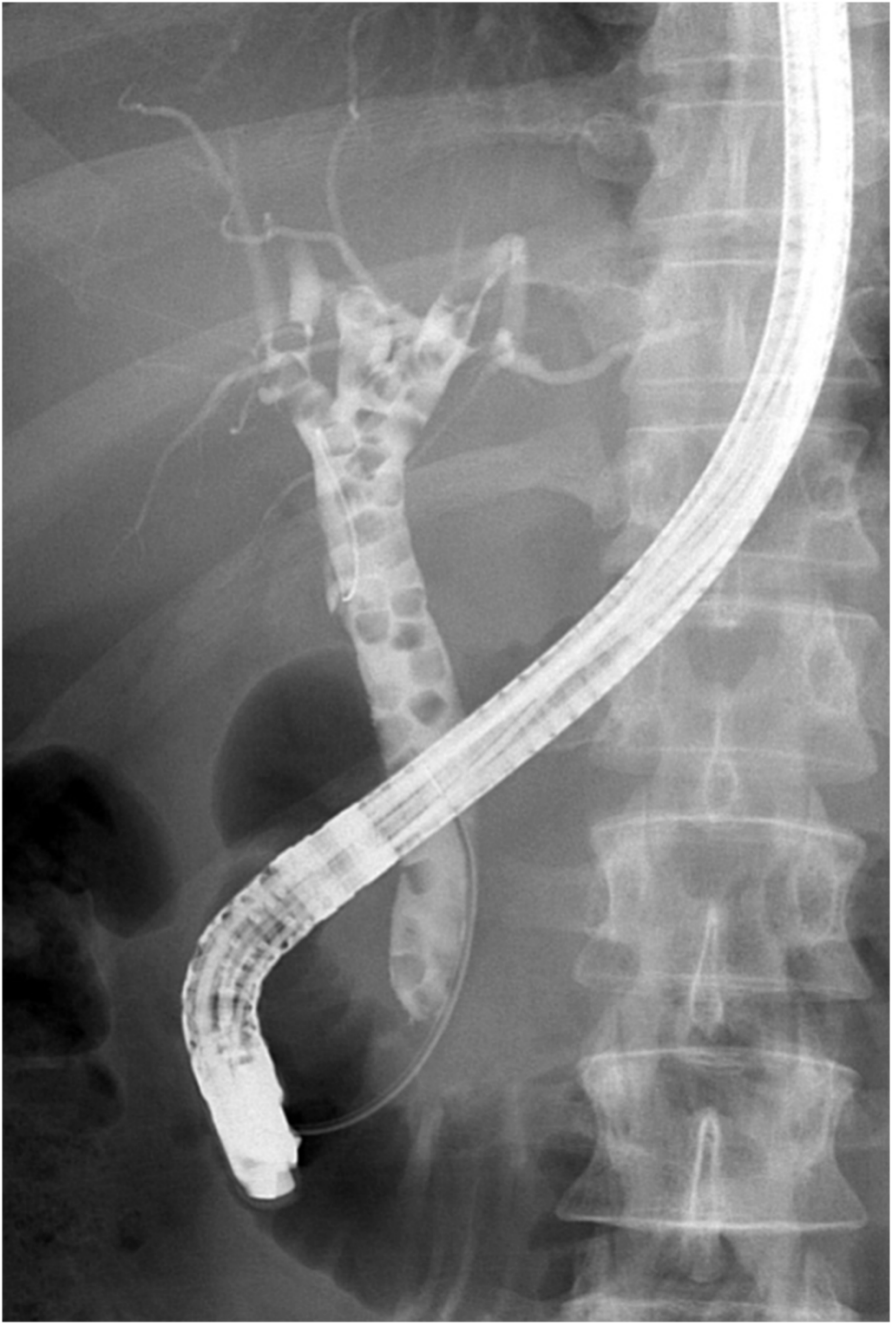
Endoscopic retrograde cholangiopancreatography showed the presence of multiple filling defects filling the entire common bile duct, common hepatic duct, and right and left hepatic ducts.

**Figure 3.**
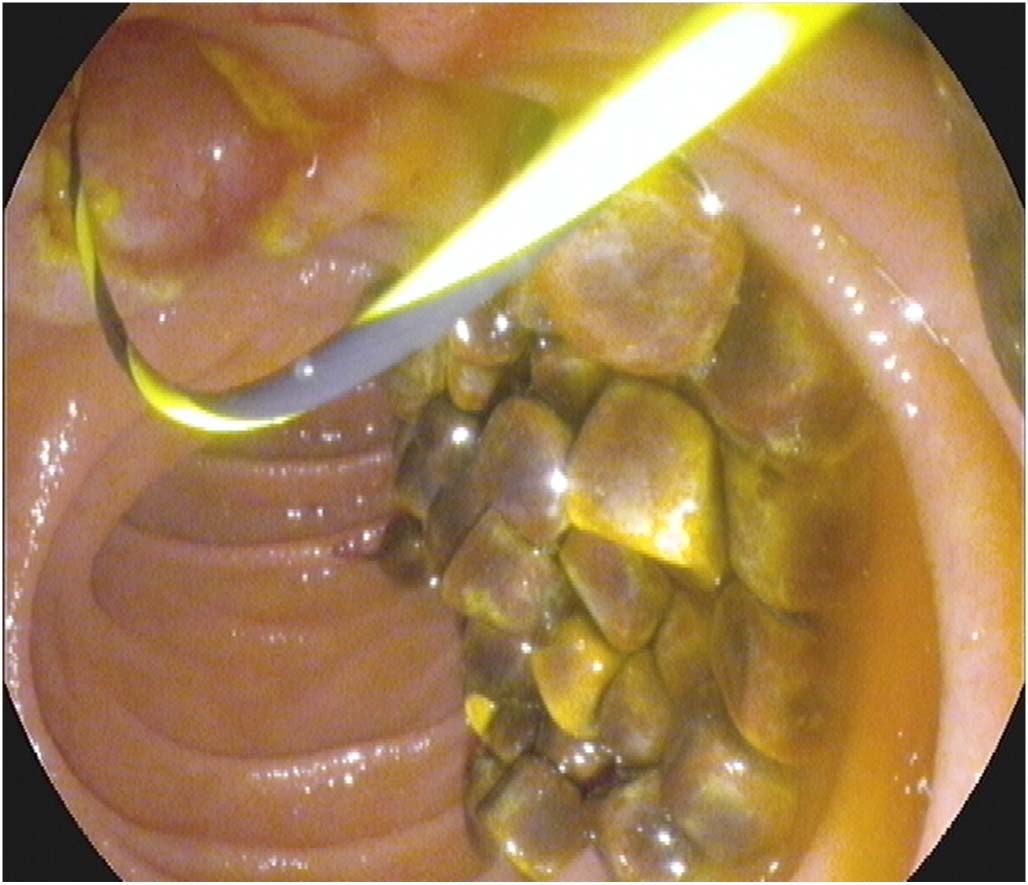
Sphincterotomy using an extraction balloon was used to remove around 100 faceted stones to clear the duct.

A 46-year-old healthy man presented with recurrent epigastric pain and nausea of 1-month duration. He denied other gastrointestinal symptoms. Physical examination was unremarkable. Laboratory tests showed the following abnormal liver enzymes: SGPT 460 IU/L, SGOT 332 IU/L, and alkaline phosphatase 462 IU/L. Magnetic resonance cholangiopancreatography showed the gallbladder, common bile duct (CBD), common hepatic duct, and proximal right and left hepatic ducts packed with gallstones, with a dilated CBD measuring 12 mm (Figure 1). Endoscopic retrograde cholangiopancreatography was subsequently performed and confirmed the presence of multiple filling defects ranging in size from 2 to 9 mm filling the entire common bile duct, common hepatic duct, and right and left hepatic ducts (Figure 2). A 5-mm sphincterotomy was performed followed by ampullary dilation with a 12-mm through-the-scope balloon. Using a 15-mm extraction balloon, 20 sweeps were required to remove around 100 faceted stones and clear the duct (Figure 3). Occlusion cholangiogram confirmed clearance of the biliary tree. The stones were too small to cause any bowel obstruction and were left to be naturally expelled.

Finally, a plastic stent was placed in the common bile duct. The patient underwent a laparoscopic cholecystectomy the next day and was discharged home the day after with an uneventful hospital stay. The management of large and/or multiple CBD stones requires usually endoscopic sphincterotomy followed by balloon dilation.^1^ Biliary stenting is associated with a reduction in the number and size of CBD stones.^2^ Studies have identified multiple potential gene mutations responsible for gallstone formation, such as *CYP7A1*, *ABCB4*, and *AOPE*.^3^ Their role in promoting gallstone development in humans is still not fully understood. The patient has no obvious risk factors or family history of gallstone disease. A genetic workup was not performed. We report a rare case of numerous gallstones filling the biliary tree. To the best of our knowledge, no other similar case was previously reported in the literature.

## DISCLOSURES

Author contributions: T. Nammour and M. Shmais wrote the article. A. Soweid edited the article and revised the article for intellectual content. T. Nammour is the article guarantor.

Financial disclosure: None to report.

Informed consent was obtained for this case report.
